# Suppression of AMF accelerates N_2_O emission by altering soil bacterial community and genes abundance under varied precipitation conditions in a semiarid grassland

**DOI:** 10.3389/fmicb.2022.961969

**Published:** 2022-08-08

**Authors:** Junqin Li, Bo Meng, Xuechen Yang, Nan Cui, Tianhang Zhao, Hua Chai, Tao Zhang, Wei Sun

**Affiliations:** ^1^Key Laboratory of Vegetation Ecology, Ministry of Education, Institute of Grassland Science, Northeast Normal University, Changchun, China; ^2^Institute of Ecology, College of Urban and Environmental Science, and Key Laboratory for Earth Surface Processes of the Ministry of Education, Peking University, Beijing, China; ^3^Key Laboratory of Mollisols Agroecology, Northeast Institute of Geography and Agroecology, Chinese Academy of Sciences, Harbin, China

**Keywords:** precipitation, AMF, N_2_O emission, bacterial community composition, functional genes

## Abstract

Nitrous oxide (N_2_O) is one of the most important greenhouse gases contributing to global climate warming. Recently, studies have shown that arbuscular mycorrhizal fungi (AMF) could reduce N_2_O emissions in terrestrial ecosystems; however, the microbial mechanisms of how AMF reduces N_2_O emissions under climate change are still not well understood. We tested the influence of AMF on N_2_O emissions by setting up a gradient of precipitation intensity (+50%, +30%, ambient (0%), −30%, −50%, and −70%) and manipulating the presence or exclusion of AMF hyphae in a semiarid grassland located in northeast China. Our results showed that N_2_O fluxes dramatically declined with the decrease in precipitation gradient during the peak growing season (June–August) in both 2019 and 2020. There was a significantly positive correlation between soil water content and N_2_O fluxes. Interestingly, N_2_O fluxes significantly decreased when AMF were present compared to when they were absent under all precipitation conditions. The contribution of AMF to mitigate N_2_O emission increased gradually with decreasing precipitation magnitudes, but no contribution in the severe drought (−70%). AMF significantly reduced the soil’s available nitrogen concentration and altered the composition of the soil bacteria community including those associated with N_2_O production. Hyphal length density was negatively correlated with the copy numbers of key genes for N_2_O production (*nirK* and *nirS*) and positively correlated with the copy numbers of key genes for N_2_O consumption (*nosZ*). Our results highlight that AMF would reduce the soil N_2_O emission under precipitation variability in a temperate grassland except for extreme drought.

## Introduction

Nitrous oxide (N_2_O) is a powerful greenhouse gas that has a global warming potential (GWP) of 298 times higher than that of carbon dioxide (CO_2_; IPCC, 2014). Doubling of concentration of N_2_O in the atmosphere would result in a 10% loss of the ozone layer, ultimately causing a 20% rise in ultraviolet radiation on the earth’s surface (Bais et al., 2018). Particularly concerning is the steep acceleration in atmospheric N_2_O concentrations over the past three decades, which from 270 parts per billion (ppb) in 1750 to approximately 331 ppb in 2018 (Tian et al., 2020). Grassland is one of the primary sources of atmospheric N_2_O, contributing to more than 30% of global emissions and accounting for global warming (Chang et al., 2021; Du et al., 2021). Although a large number of studies have investigated the contribution of grassland ecosystems to atmospheric N_2_O emissions, the mechanisms of grassland N_2_O emission under climate change are still not well understood (Li et al., 2020; Chang et al., 2021).

The production of soil N_2_O is controlled by various biotic and abiotic factors. Previous studies have provided a comprehensive description of the abiotic factors that affect the production of soil N_2_O, including soil moisture, soil temperature, soil aeration, pH, C/N ratio, and soil texture (Kumar et al., 2020). Global climate change, such as extreme droughts and extreme precipitation events, can have a significant impact on these biotic and abiotic factors (Sheffield et al., 2012; IPCC, 2014), which may accelerate or slow down ecosystem soil N_2_O emission processes. Generally, N_2_O emissions show a nonlinear growth relationship with soil moisture status, with the highest N_2_O production in modest soil water content, whereas, the lowest occurred under saturated and arid soils (Kumar et al., 2020). However, the response of soil N_2_O emissions to the changes in rainfall and its potential mechanisms is still not very clear. Furthermore, soil N_2_O emissions are the result of microbial processes, with more than 60% of N_2_O emissions occurring from nitrification and denitrification by-products (Ishii et al., 2011; Signor and Cerri, 2013). The sensitivity of soil microorganisms to environmental factors (soil water status) and the variability of future climate (precipitation magnitudes) lead to difficulties in predicting the impact of climate change on soil N_2_O emissions.

Arbuscular mycorrhizal fungi (AMF), one of the major important components of the soil microorganisms, can form mutualistic associations with more than 72% of terrestrial plant species (Brundrett and Tedersoo, 2018), and it is now widely recognized that AMF plays a vital role in soil N-cycling processes (Govindarajulu et al., 2005; Veresoglou et al., 2012; Storer et al., 2018). A growing body of research have shown that AMF could reduce the emission of soil N_2_O (Bender et al., 2014; Storer et al., 2018; Okiobe et al., 2019). Bender et al. (2014) attributed the reduction in N_2_O emissions by AMF to the fact that these fungi reduce N_2_O emission substrates by facilitating the assimilation of soil N by plants and microbes. Moreover, similar studies have shown that AMF would indirectly affect denitrification to alleviate soil N_2_O emissions (Okiobe et al., 2019). AMF-induced variations in the soil microbial community determine the abundance of key genes that are responsible for N_2_O production (*nirK* and *nirS*) and consumption (*nosZ*), which ultimately regulate N_2_O emissions (Bender et al., 2014; Waghmode et al., 2018). However, the contribution of AMF to the reduction in N_2_O emissions might not be consistent in the different ecosystems, and whether there is a drought threshold that could alter the AMF effect on N_2_O emissions remains unclear.

To explore the effects of AMF on N_2_O emissions under varied precipitation conditions, we conducted an *in situ* experiment with two factors, precipitation gradient and AMF suppression in a semiarid grassland in northeastern China. We addressed the following three hypotheses: (1) Precipitation magnitudes would be closely related to N_2_O emission, soil moisture content would be positively correlated with N_2_O emission; (2) AMF could alleviate soil N_2_O emission by changing soil properties, the bacterial community composition and N cycle-related functional gene abundance under the different precipitation intensities; (3) There may be a nonlinear response pattern in the contribution of AMF to the reduction in soil N_2_O emissions with decreasing soil water content gradient.

## Materials and methods

### Experimental site

The study site was located at the Jilin Songnen Grassland Ecosystem National Observation and Research Station (44°40′–44°44′ N, 123°44′–123°47′ E; 160 m above sea level) of the Northeast Normal University, Jilin Province, northeastern China. The study site has a temperate semiarid monsoon climate with mean annual temperature and precipitation ranging from 3.4°C–7.6°C and 258–716 mm (1953–2017), respectively. Approximately 70% of precipitation occurs during the vigorous plant growing season (June–August). The experimental site vegetation is dominated by the C_3_ perennial rhizomatous grass *Leymus chinensis* (over 90% of plant cover), other accompanying species include perennials grasses (*Phragmites australis* and *Hemarthria altissima*) and annuals grasses, such as *Chloris virgate* (Zhong et al., 2017; Mei et al., 2019; Yang et al., 2021). Our previous work showed that the main taxon of arbuscular mycorrhiza fungi in the genus *Glomus* in this area (Zhang et al., 2016). The main soil type of semiarid grassland is chernozem with a pH of 8.0–9.0, soil total nitrogen content of 0.15%, and total organic carbon content of 2.0%. Soil texture is 35% clay, 45% silt, and 20% sand on average. Bulk density is 1.44 g cm^−3^, and field capacity is approximately 0.255 g g^−1^ (Meng, Ochoa-Hueso et al., 2020; Meng, Li et al., 2021).

### Experimental design

The precipitation manipulation experiment was established in 2015 [for details, see Yang et al. (2021)]. Specifically, we fenced a 1 ha area (100 × 100 m) of grassland and divided split it equally into four blocks (25 × 25 m). In each block, six plots (3.5 × 3.5 m) were subdivided, with a buffer zone greater than 2 m between plots. Six plots within a block were randomly assigned to one of six precipitation treatments: increase 50% (+50%), increase 30% (+30%), ambient (0%), decrease 30% (−30%), decrease 50% (−50%), and decrease 70% (−70%), replicated four times and a total 24 plots. The rainout shelters were installed in each plot to create the precipitation gradient [for details see Li et al. (2019)]. After each rainfall event, the intercepted rainfall from a shelter in the −30% and −50% plots were irrigated to the +30% and +50% plots by manual spraying, respectively. Each plot used water-blocking plates (stainless steel: 0.5 m belowground and 0.15 m aboveground) around the plots to avoid water from overland runoff and belowground lateral soil infiltration. We used a control treatment (without rainout shelters, 0%) to identify that our rainout shelters have no impact on plant photosynthesis (Li et al., 2019) and soil properties (Yang et al., 2021).

*In situ* AMF treatment was manipulated by modifying the method of growth cores described by Johnson et al. (2001) and Li et al. (2019). The cores were constructed using a PVC (polyvinyl chloride) tube (height 20 cm and inner diameter 11 cm), where approximately 50% of the surface area was removed and sealed with a 35 μm mesh to allow the pass of water and AMF mycelium. Through a repeated slight rotation of this core [after rotation, soil from the plots and sieved (1 mm) was used to fill the gap between the PVC pipe and the soil], we reduced AMF growth in it. This approach allows testing the effect of localized reduction in AMF abundance within field plots, without potential indirect effects such as changes in plant growth and exudation levels that may occur in response to the soil microenvironment. On 21 May 2019, 2.2 kg of soil from each plot was loaded into the cores (sieving to remove stones and gravel) and these soil-filled cores were randomly installed in the plots where the soil was collected (0.5 m from the water-blocking plate to reduce edge effects). Every 2 days, we rotated half of all cores per plot approx. 45° around their vertical axes to break AMF hyphae penetrating the core (AMF-excluded). The remaining half was kept stationary, allowing mycelium to penetrate the core (AMF-permitted).

### Precipitation, air temperature, and soil moisture content

Climate data, including precipitation and air temperature, were continuously monitored using the RG2-M sensor (Oneset Computer Corporation, Bourne, MA, United States) for the entire 2019–2020 growing season. Soil water content (SWC) monitoring sensors (S-TMB-M005, Decagon, Pullman, WA, United States) were placed at 0–10 cm of soil, and the sensors automatically recorded data every 30 min. In addition, soil water content during the growing season was tested by oven-drying soil samples from 0 to 10 cm of each plot (May–September), once a month.

### Sampling and measurement of nitrous oxide

Soil N_2_O emissions in the AMF cores were measured every 15 days from June to August in 2019 and 2020 by using the closed static chamber technique. Gas was collected between 08:30 and 11:00 am, using a custom-made cylindroid acrylic chamber (45 cm in inner height and 11 cm in inner diameter, covered with aluminum foil). The bottom of the chamber was encased with a rubber ring for an air-tight seal when the chamber was installed. To facilitate the gas collection, we punched a 2.5 mm diameter hole in each lid and tightly fitted a gas check valve that could be connected to the chamber. During a pre-experiment, gas samples were collected 0, 15, 30, 45, and 60 min after chamber closure. The results showed that the N_2_O concentration in the chamber increased linearly with time during the first 0–45 min (*R*^2^ > 0.9). Therefore, during the experiment, we collected two gas samples at 0 and 40 min using an injection syringe, respectively. The temperature inside and outside the chamber was recorded simultaneously with a thermometer. The concentration of N_2_O was assessed using a nitrous oxide/methane analyzer (Model 913–1,054, Los Gatos Research, United States). The N_2_O flux was calculated by the following formula:


$$f_{N_{2}O}=\frac{\left(c_{2}\times V\times M_{0}\times \frac{273}{273+T_{2}}\right)-\left(c_{1}\times V\times M_{0}\times \frac{273}{273+T_{1}}\right)}{A\times t\times 22.4\times 10^{-3}}$$

where *f_N2O_* is the flux of N_2_O (μg m^−2^ h^−1^); *c_1_* and *c_2_* are the concentrations of N_2_O at 0 min and 40 min in the static closed chambers, respectively (μg m^−2^); *V* is the volume of the chamber (m^3^); *M_0_* is the molar mass of N_2_O; *T_1_* and *T_2_* are the temperatures at 0 min and 40 min in the static closed chambers, respectively (°C); *A* is the area of the bottom of the chamber (m^2^); and *t* is the time of gas collection (h).

The accumulative N_2_O emission throughout the experiment period was calculated by the following formula:


$$F_{N_{2}O}=\overset{n}{\underset{1}{\sum }}\left(\frac{\left(d_{i+1}-d_{i}\right)\left(f_{i+1}+f_{i}\right)}{2}\times 24\right)\times \frac{A}{1000}$$

where *F_N2O_* (mg m^−2^) is the accumulation of N_2_O; *d_i + 1_* − *d_i_* is the date of the interval between two gas collections; *f_i + 1_ + f_i_* is the sum of the fluxes of the two gas collections, *A* is the area of the bottom of the static chamber.

### Soil sample and mycorrhizal hyphae analysis

An amount of 100 g soil sample (0–15 cm) was collected from AMF cores using 2 cm diameter soil cores on August 25, 2019 (backfilled with identical sterile soil) and August 26, 2020. Soil pH was measured by using a combination glass electrode in a 1:2.5 soil–water mixture suspension. Ammonium nitrogen (NH_4_^+^-N) and nitrate-nitrogen (NO_3_^−^-N) concentrations were analyzed by using a continuous flow analyzer (Futura II, Alliance Instruments Ltd., Frépillon, France) in leaching extraction of 1:5 soil and KCl solution (2 M). The soil net nitrification rate (NR) and net mineralization rate (MR) were measured during aerobic incubation according to Hart et al. (1994). An amount of 10 g (equivalent dry mass) of fresh soil were placed in a 100 ml glass flask, which was then sealed with a sealing film. The soil was incubated for 15 days at 25°C in the dark, and then the NO_3_^−^ and NH_4_^+^ concentrations were measured. NR and MR were determined as the difference in NO_3_^−^ and inorganic-N between initial and incubated samples, respectively. Total nitrogen (TN) was analyzed by using an elemental analyzer (vario EL cube, Elementar, Langenselbold, Germany).

The mycorrhizal hyphae development in the cores was assessed in the cores according to the method described by Jakobsen et al. (1992). Briefly, the extraradical hyphae of AMF were extracted by filter membrane extraction, stained with 0.05% trypan blue, and 25 fields of view were randomly observed at 200× microscopes and the number of mycelial crossover points was recorded using the gridline intercept method. The length of extraradical mycelium per unit dry weight (g) was used to reflect the density of extraradical mycelium in the soil samples, called hyphal length densities (HLD, m hyphae g^−1^ soil dry weight).

### Bacterial community composition and quantitative PCR analysis

In this study, 16S rRNA amplicon sequencing was performed using the Illumina HiSeq platform to assess the effects of precipitation and AMF suppression on the soil bacterial community. DNA was extracted using the MN NucleoSpin 96 Soil kit (MN, Germany) according to the instructions provided by the manufacturer. The V3-V4 region of the 16S rRNA gene was amplified in triplicate using the extracted DNA as a template and the primer pairs and reaction conditions are shown in Supplementary Table S1. PCR reactions were performed in triplicate using an Applied Biosystems ProFlex 2 × 96-well PCR instrument (9,902, ABI, United States).

To test the microbiological mechanism of AMF affects N_2_O emission, we quantified the copy numbers of key genes involved in N_2_O production and consumption (nitrification and denitrification) in the core soil, which encode cd1 and copper nitrite reductases (*nirS* and *nirK*), nitrous oxide reductase (*nosZ*) and associated with ammonia oxidation (AOA and AOB; Zumft, 1997; Gui et al., 2021). Gene copy number estimations were performed using relative real-time estimation against a reference target (16S rRNA) to increase the accuracy and sensitivity of detection (Daniell et al., 2012). All functional gene amplifications were performed in triplicate using a LightCycler480 II Real-Time PCR System (Roche, Rotkreuz, Switzerland), with three technical replicates per sample, with the primer pairs and reaction conditions shown in Supplementary Table S2.

### Statistical analyses

Mycorrhizal responses (R%) of accumulative N_2_O emission were calculated using the individual values of AMF-permitted and mean values of AMF-excluded within each treatment.


$$R\%=\frac{\mathrm{AMF-permitted}-\mathrm{mean}\;\mathrm{AMF-excluded}}{\mathrm{mean}\;\mathrm{AMF-excluded}}\times 100$$

For all data, the normality of the variance was checked using the Kolmogorov–Smirnov criterion before performing ANOVA. We used two-way ANOVA to test the effects of precipitation, AMF suppression, and their interaction on HLD, available N, N cycle-related gene copy number (AOA, AOB, *nirK*, *nirS*, and *nosZ*) and N_2_O emission. One-way ANOVA followed by Tukey *post-hoc* tests was used to further assess differences in the SWC, HLD, available N, N cycle-related gene copy number, N_2_O emission, and the contribution of mycorrhizal on N_2_O emission between different AMF treatments under the different precipitation conditions. The significance of treatment effects between AMF-permitted and AMF-excluded was assessed using a *T*-test at *p* < 0.05. The results were expressed as the mean value ± standard error (SE, *n* = 4). Pearson correlation analysis was used to exploit the relationship of SWC and N_2_O flux, HLD and accumulative N_2_O emissions, respectively.

The bacteria diversity (B_Simpson) and richness (B_OTUs) were calculated based on the OTU level of bacteria through the vegan package in R software (Version 3.6.0). The relative abundance of the bacterial phylum under different treatments was displayed by a stacked bar chart using the ggplot2 package. The overall relationship between the N cycle-related factors (HLD, SWC, pH, NO_3_^−^-N, NH_4_^+^-N, NR, and N_2_O emission) and the top 20 abundance of bacterial genus taxa across all samples were visualized by the psych heatmap in R. Considering the realistic level of microbial diversity in this study, we analyzed the difference of species abundances between AMF-permitted and AMF-excluded at the genus level. A correlation matrix including two growing seasons in 2019 and 2020 was constructed to look for relationships between soil properties (SWC, pH, NO_3_^−^-N, NH_4_^+^-N, NR, MR, and Soil TN), HLD, bacterial community composition (B_OTUs and B_Simpson), N cycle-related gene copy number (AOA, AOB, *nirK*, *nirS*, and *nosZ*), and N_2_O emission.

Structural equation modeling (SEM) was used to study the direct and indirect of precipitation variation and AMF on N_2_O emission. The AMF variable was an indication of the hyphal length density (HLD) of the soil AM fungus. The available N was the soil inorganic N content, which was the sum of ammonium N and nitrate N. Our structural equation modeling was carried out using the SEM function of the lavaan package in R software (version 3.4.3). We used three different metrics as in Wang et al. (2020) to determine the goodness of fit of our model: the Chi-square test (*χ*^2^; 0 ≤ *χ*^2^ ≤ 2df and *p* > 0.05 indicating a good fit of the model), Bentler’s comparative fit index (CFI; CFI > 0.95 indicating a good fit of the model) and the standardized root means square residual (SRMR; SRMR ≤ 0.08 indicating a good fit of the model).

## Results

### Climate variation and soil microclimate

**Figure 1:**
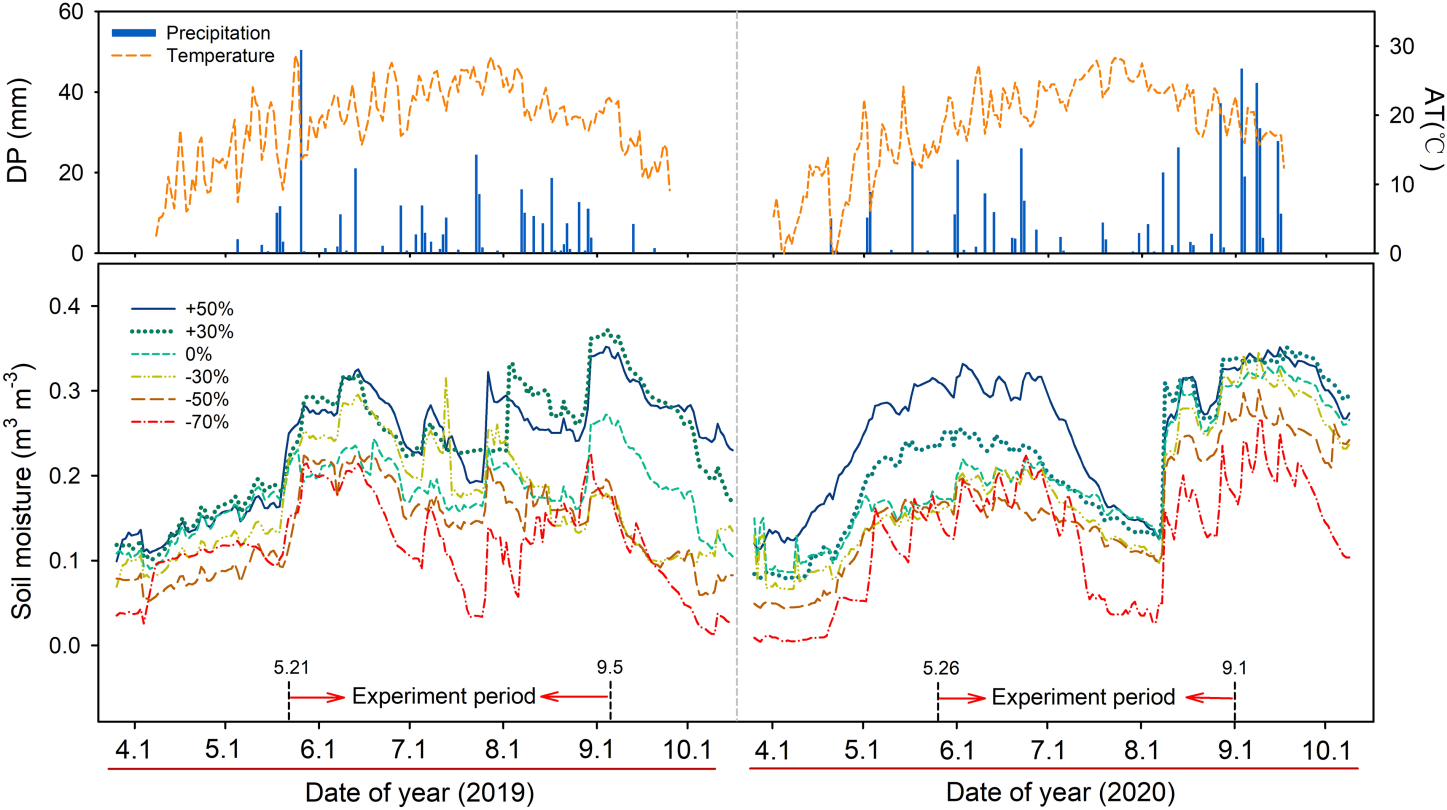
Variation in daily precipitation (DP, mm), air temperature (AT, °C), and soil water content (*n* = 4, m^3^ m^−3^) at 10 cm soil depth for the six precipitation treatments in 2019 and 2020.

The growing season precipitation (May–September) was 320.4 mm and 479.6 mm in 2019 and 2020, respectively. Air temperature showed seasonal dynamics in both years, with the highest temperature in July of each year (Figure 1). Soil moisture was influenced by precipitation treatments, which declined sequentially with the decrease in precipitation. Significant main effect of precipitation gradient on soil water content (SWC, *p* < 0.05) was observed across the two growing seasons (Supplementary Figure S1).

### The effects of precipitation and AMF on N_2_O emission

The soil N_2_O emissions were significantly influenced by precipitation and AMF. N_2_O fluxes and cumulative emission decreased significantly with the decline of precipitation during the peak growing season (June, July, and August) in 2019 and 2020 (all *p* < 0.05, Supplementary Figure S2, Table 1). The average N_2_O flux during the peak of the growing season in 2019 and 2020 decreased by 133.6% and 172.8% with precipitation from +50% to −70%, respectively, while leading to a decrease in accumulative N_2_O emissions decreased by 134.6% and 186.5%, respectively. In addition, N_2_O fluxes of AMF-excluded treatment in both 2019 and 2020 were significantly higher than AMF-permitted treatment across all precipitation conditions, with a relatively average increase of 44.7% (*p* < 0.05) and 30.0% (*p* < 0.05), respectively (Table 1).

**Table 1:** N_2_O fluxes (μg m^−2^ h^−1^) and soil available N concentration under different precipitation treatments in 2019 and 2020.

			**2019**						**2020**					
			**+50%**	**+30%**	**0%**	**−30%**	**−50%**	**−70%**	**+50%**	**+30%**	**0%**	**−30%**	**−50%**	**−70%**
**N**_**2**_**O flux (μg m**^**−2**^ **h**^**−1**^**)**	Jun.	AMF-p	36 ± 5**b**	32 ± 6**b**	27 ± 7**b**	16 ± 3**b**	18 ± 8**a**	16 ± 6**a**	48 ± 10**a**	49 ± 3**a**	40 ± 6**b**	38 ± 10**b**	24 ± 11**b**	24 ± 3**a**
		AMF-e	58 ± 7**a**	62 ± 8**a**	60 ± 6**a**	70 ± 7**a**	28 ± 7**a**	20 ± 4**a**	67 ± 3**a**	57 ± 4**a**	61 ± 13**a**	51 ± 10**a**	44 ± 7**a**	34 ± 4**a**
	Jul.	AMF-p	51 ± 8**b**	33 ± 9**a**	28 ± 4**b**	22 ± 2**b**	14 ± 3**b**	26 ± 1**b**	17 ± 2**a**	17 ± 4**a**	16 ± 1**a**	10 ± 2**a**	8 ± 3**a**	5 ± 1**a**
		AMF-e	80 ± 8**a**	47 ± 9**a**	55 ± 7**a**	46 ± 8**a**	34 ± 4**a**	30 ± 2**a**	23 ± 3**a**	20 ± 2**a**	18 ± 6**a**	17 ± 4**a**	14 ± 5**a**	5 ± 2**a**
	Aug.	AMF-p	35 ± 3**b**	35 ± 5**b**	27 ± 4**b**	31 ± 5**b**	25 ± 5**b**	24 ± 4**a**	30 ± 7**a**	30 ± 8**b**	24 ± 2**b**	27 ± 5**b**	20 ± 2**a**	6 ± 3**a**
		AMF-e	62 ± 9**a**	70 ± 11**a**	54 ± 7**a**	47 ± 5**a**	51 ± 7**a**	22 ± 5**a**	42 ± 5**a**	42 ± 1**a**	40 ± 2**a**	42 ± 12**a**	30 ± 4**a**	8 ± 1**a**
**Available N (mg kg**^**−1**^ **soil)**	NH_4_^+^	AMF-p	3.9 ± 0.8**a**	4.4 ± 0.2**a**	4.2 ± 0.7**a**	2.6 ± 0.2**a**	2.4 ± 0.2**a**	1.3 ± 0.3**a**	1.6 ± 0.2**a**	1.8 ± 0.5**a**	2.1 ± 0.2**a**	1.7 ± 0.2**a**	1.4 ± 0.3**a**	1.4 ± 0.2**a**
		AMF-e	2.4 ± 0.6**a**	3.0 ± 0.3**b**	3.2 ± 0.1**a**	1.8 ± 0.1**b**	1.6 ± 0.2**b**	1.5 ± 0.3**a**	1.6 ± 0.3**a**	1.6 ± 0.2**a**	1.7 ± 0.2**a**	1.2 ± 0.2**a**	1.1 ± 0.1**a**	1.3 ± 0.1**a**
	NO_3_^−^	AMF-p	9 ± 0.3**b**	11 ± 0.4**b**	12 ± 1**b**	11 ± 1**b**	12 ± 2**a**	17 ± 2**b**	1 ± 0.4**b**	3 ± 0.3**a**	2 ± 0.6**b**	7 ± 0.3**a**	9 ± 1**a**	10 ± 2**b**
		AMF-e	11 ± 0.3**a**	18 ± 2**a**	19 ± 0.7**a**	20 ± 1**a**	27 ± 3**a**	24 ± 1**a**	6 ± 1**a**	6 ± 1**a**	8 ± 0.6**a**	8 ± 0.6**a**	12 ± 1**a**	17 ± 1**a**

Lowercase letters indicate significant differences (*p* < 0.05) between AMF-permitted (AMF-p) treatment and AMF-excluded (AMF-e) treatment in difference precipitation conditions. Data are reported as mean ± 1 SE (*n* = 4).

**Figure 2:**
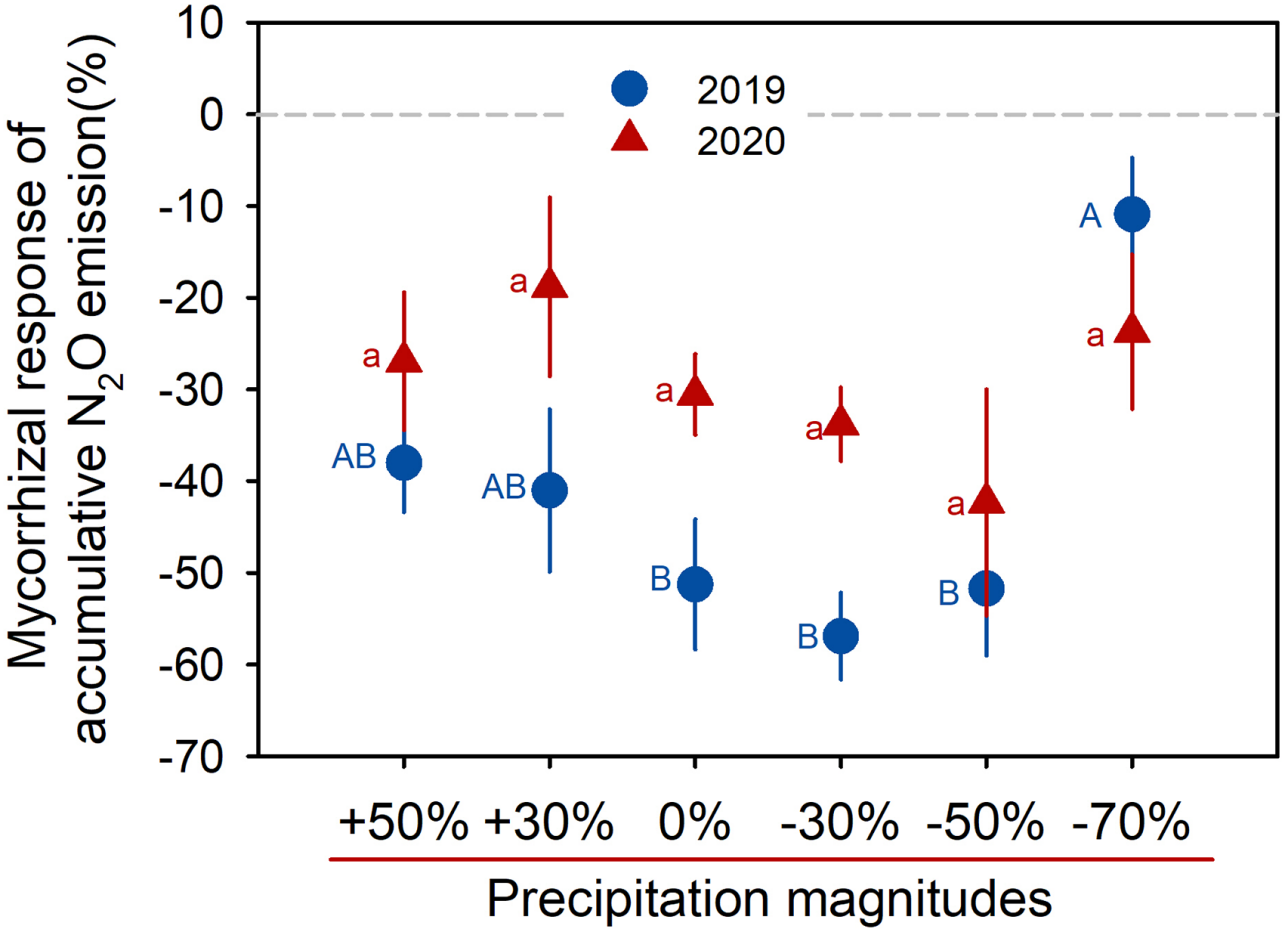
Mycorrhizal response of accumulative N_2_O emission for six precipitation treatments in 2019 and 2020. Data are reported as mean ± 1 SE (*n* = 4). Different lowercase and capital letters indicate significant differences (*p* < 0.05) among the precipitation treatments in 2019 and 2020, respectively.

**Figure 3:**
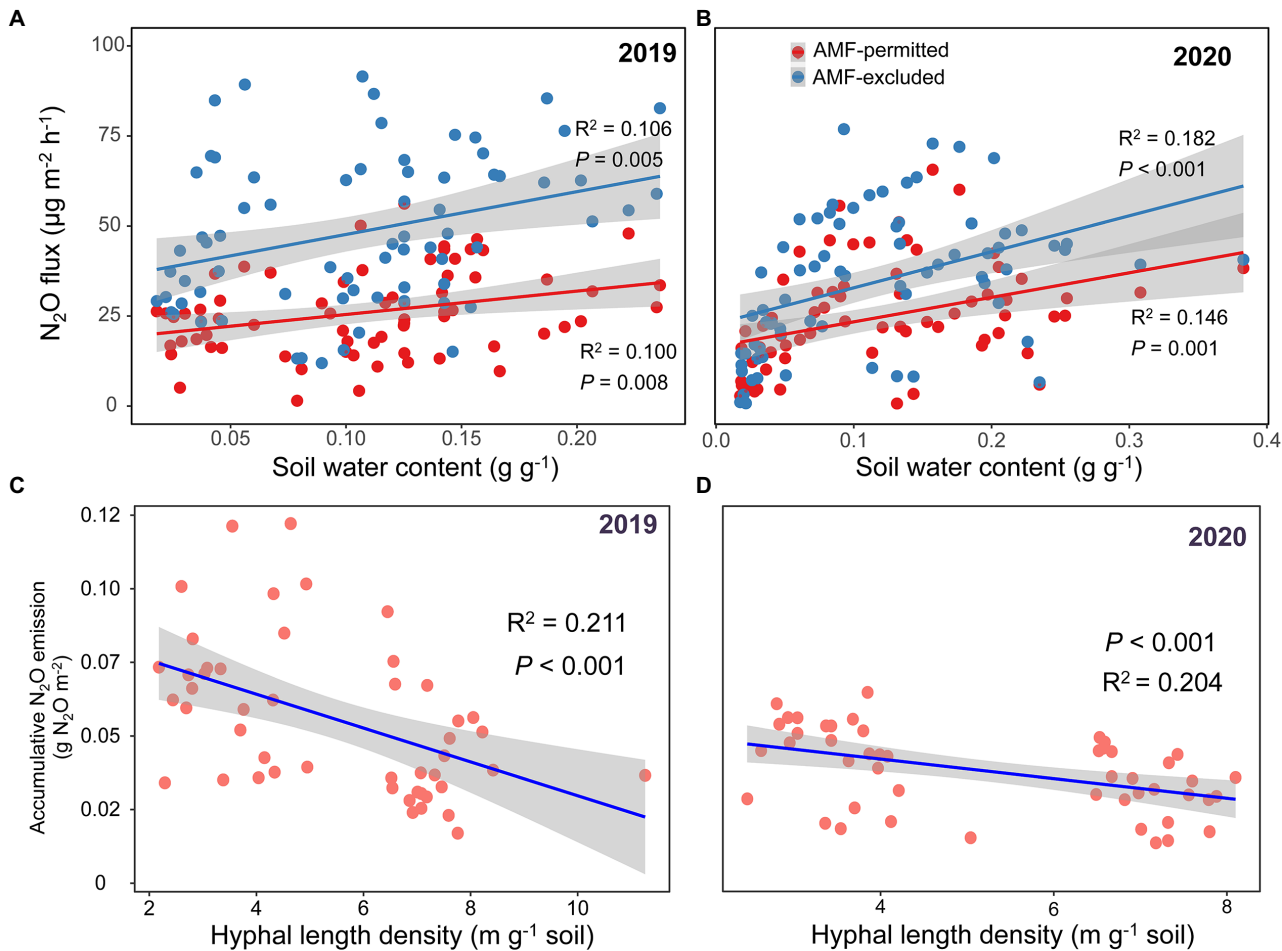
Relationships between soil water content (SWC) and N_2_O flux across growing seasons in both 2019 **(A)** and 2020 **(B)**. Relationships between hyphal length density and accumulative N_2_O emission across growing seasons both 2019 **(C)** and 2020 **(D)**. The fitted lines are from the OLS regression. Shaded areas show a 95% confidence interval of the fit.

The mycorrhizal response of accumulative N_2_O emission showed that AMF favored mitigation of soil N_2_O emissions (values <0) under all precipitation treatments in both growing seasons, and the response was gradually increased with precipitation gradient reduction, but sharply decreased in the −70% treatment (Figure 2). Significant positive correlations between soil water content and N_2_O fluxes were detected across all AMF treatments (all *p* < 0.05, Figures 3A,B), and the HLD was negatively correlated with accumulative N_2_O emissions in 2019 and 2020 (both *p* < 0.001, Figures 3C,D).

### Effect of AMF on soil available N, soil bacteria community composition, and N cycle functional genes abundance under precipitation change

The decrease in precipitation magnitude significantly reduced NH_4_^+^-N concentrations and increased NO_3_^−^-N concentrations in two growing seasons. Compared to AMF-permitted, AMF-excluded significantly decreased NH_4_^+^-N concentrations in 2019 (*p* < 0.05), but no impact in 2020 (*p* > 0.05), and remarkably increased NO_3_^−^-N concentrations in both 2019 and 2020 (all *p* < 0.05, Table 1).

**Figure 4:**
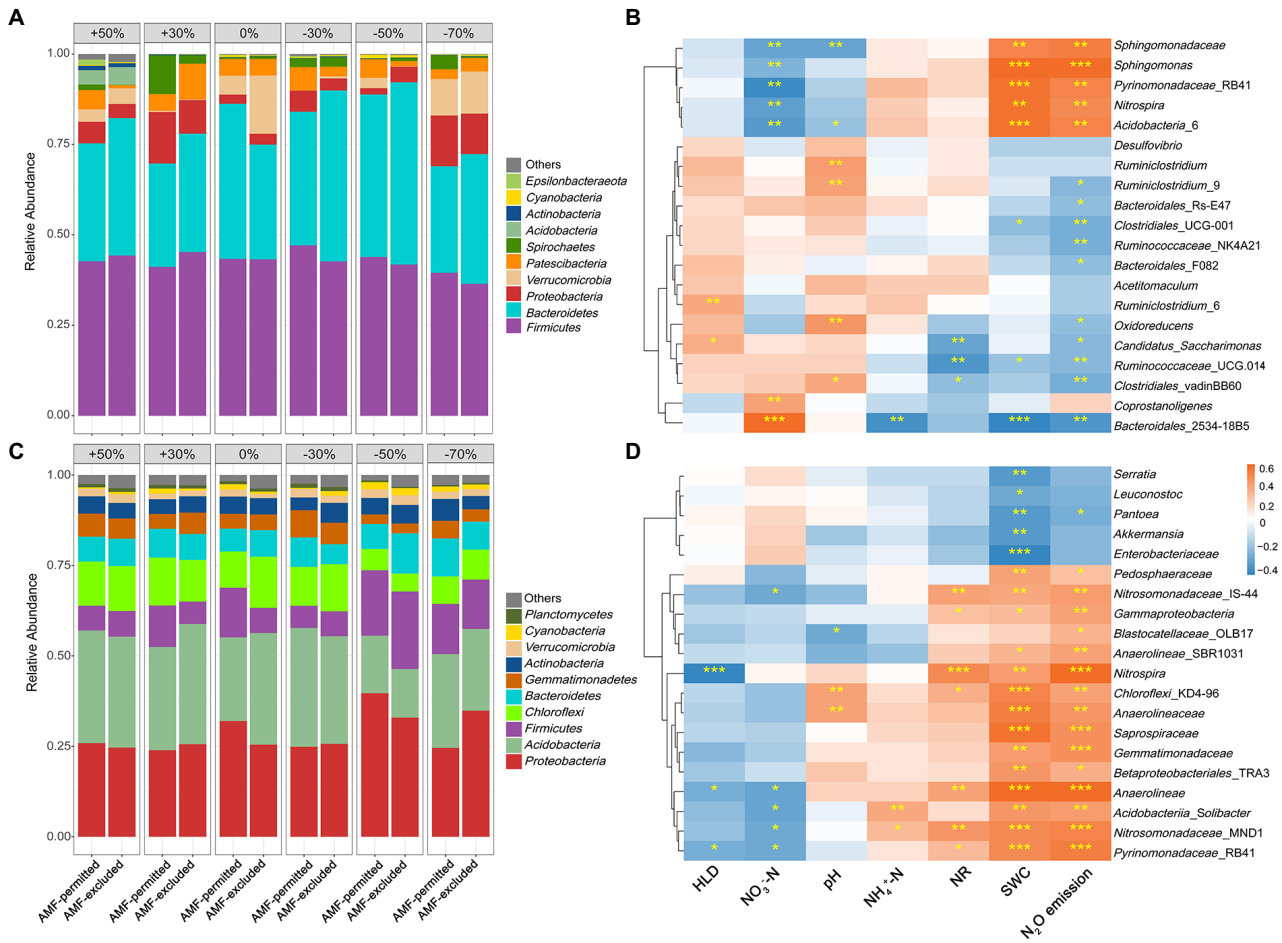
Phylum-level microbial composition of soils under AMF-permitted and AMF-excluded in different precipitation conditions in 2019 **(A)** and 2020 **(C)**. Heatmap of correlation of Bray-Curtis distances of the microbial abundance and soil properties at the level of operational taxonomic units (97% DNA sequence identity) for 2019 **(B)** and 2020 **(D)**.

Both precipitation and AMF suppression altered soil bacterial community composition. With the decrease in precipitation magnitude, the relative abundances of *Bacteroidetes* and *Proteobacteria* increased in both years, and the relative abundance of *Firmicutes* decreased in 2019 and increased in 2020 (Figures 4A,B). AMF suppression affected the relative abundance of soil bacteria in the top 10 most abundant phyla in both growing seasons (Figures 4A,B), and significantly altered the abundance of bacteria at the genus level (*p* < 0.05, Supplementary Figure S3). Significantly negative correlations between the HLD and the abundance of genera associated with N_2_O emissions were observed (Figures 4C,D), for example, AMF-excluded increased the abundance of *Nitrospira* (Supplementary Figure S4).

**Figure 5:**
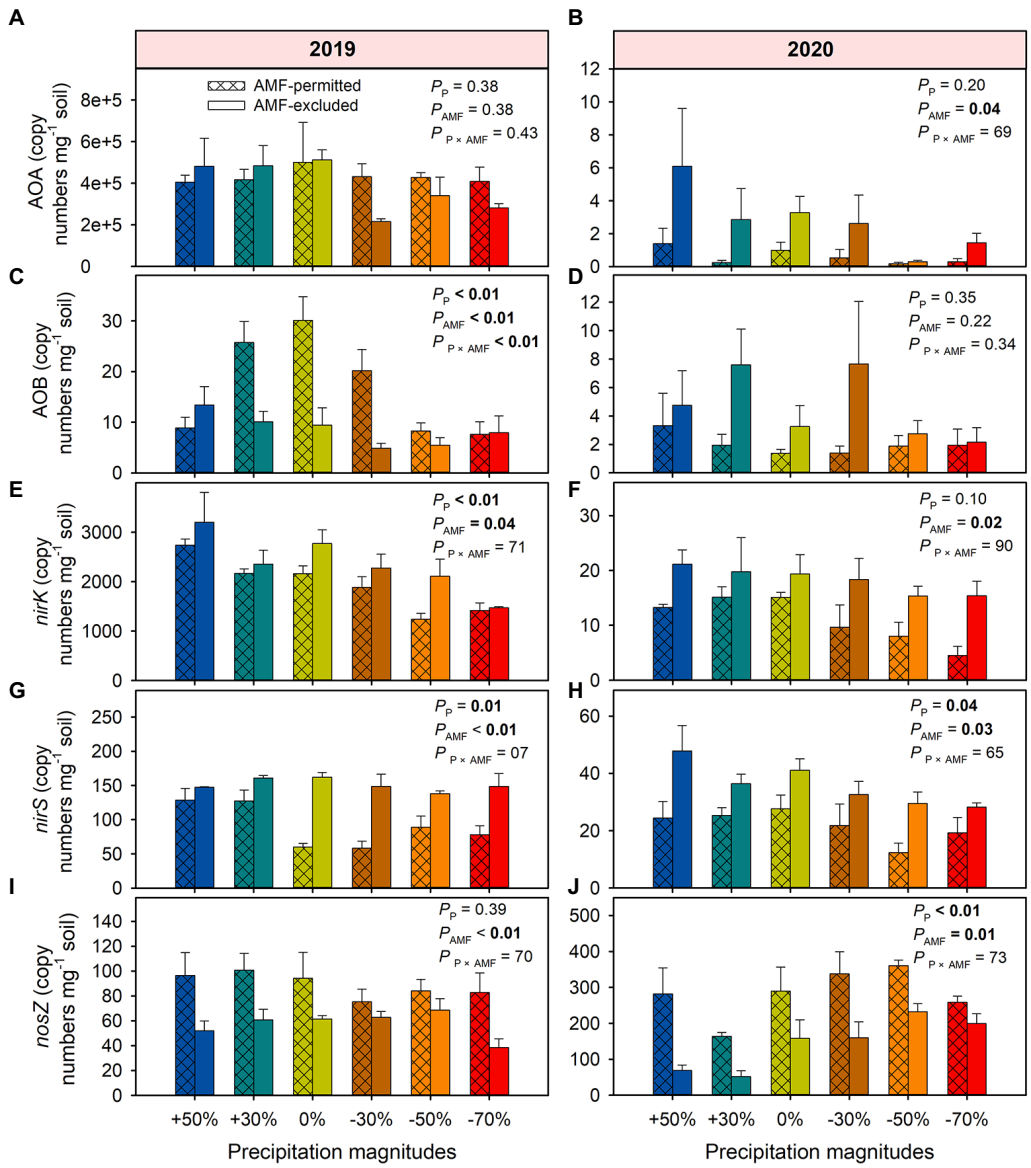
The effects of precipitation and AMF on functional gene copy numbers in 2019 **(A,C,E,G,I)** and 2020 **(B,D,F,H,J)**. The values in boldface type denote significant differences between treatments (*p* < 0.05). Data are reported as mean ± 1 SE (*n* = 4).

AMF suppression increased the copy numbers of AOA by 41% on average (*F* = 10.61, *p* = 0.04) across all treatments in 2020 but not in 2019 (Figures 5A,B). In 2019, precipitation reduce or increase had decreased the copy number of AOB compared with ambient condition when the presence of AMF in 2019, AMF-excluded significantly decreased the copy numbers of AOB by 31% on average (*F* = 116.0, *p* = 0.002, Figure 5C). Under all precipitation treatments, AMF-excluded meanly increased copy numbers of *nirK* by 25% (*F* = 12.5, *p* = 0.03) and 89% (*F* = 20.7, *p* = 0.02) in 2019 and 2020, respectively (Figures 5E,F), and increased the copy numbers of *nirS* by 85% (*F* = 25.9, *p* < 0.001) and 71% (*F* = 16.7, *p* = 0.03) on average across all precipitation treatments in 2019 and 2020, respectively (Figures 5G,H). Meanwhile, the copy numbers of *nirS* in AMF-excluded was lower by 35% (*F* = 156.2, *p* = 0.001) and 50% (*F* = 32.3, *p* = 0.01) than that in AMF-permitted in 2019 and 2020, respectively (Figures 5I,J).

**Figure 6:**
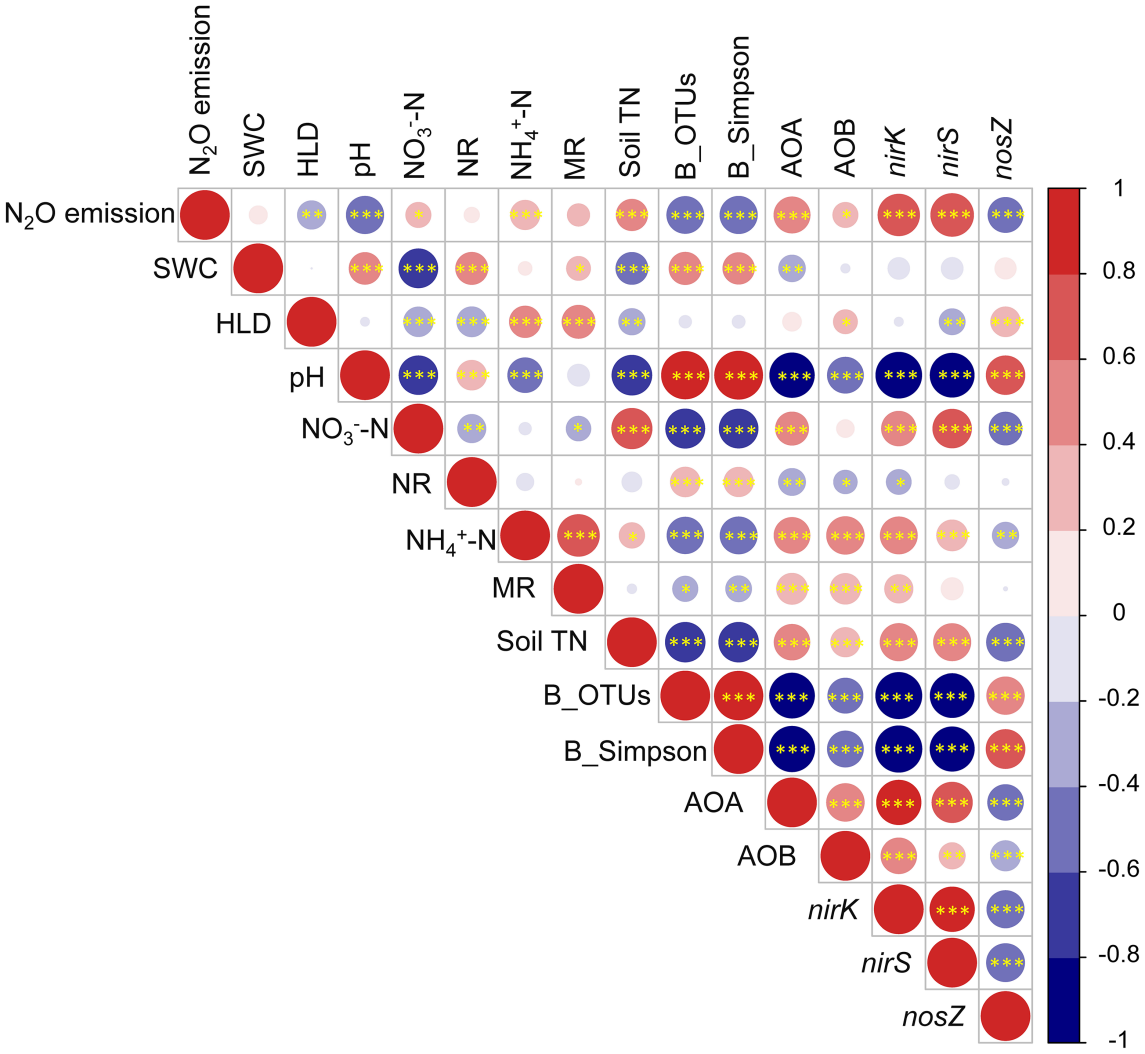
Pearson correlations among N_2_O emission, AMF (HLD), soil (SWC, pH, NO_3_^−^-N, NR, NH_4_^+^-N, MR, and TN), bacteria (OTUs, Simpson), and functional genes abundance (AOA, AOB, *nirK*, *nirS*, and *nosZ*) for both years. HLD, hyphal length density; SWC, soil water content; NO_3_^—^N, nitrate nitrogen; NH_4_^+^-N, ammonium nitrogen; NR, net nitrification rate; MR, net mineralization rate; soil TN, soil total nitrogen; B_OTUs, bacterial richness; B_Simpson, bacterial diversity; *p < 0.05, **p < 0.01, and ***p < 0.001.

Pearson correlation analysis showed that AMF, soil bacterial community composition and N cycle functional genes significantly correlated with the N_2_O emission (Figure 6). Soil water content was positively correlated with pH, net nitrification rate, net mineralization rate, and bacterial community composition, but negatively correlated with nitrate, soil total N, and AOA copy numbers. The HLD was positively correlated with ammonium, AOB copy numbers, and *nosZ* copy numbers, but negatively correlated with nitrate N, net nitrification rate, soil total N, and *nirS* copy numbers. The HLD, pH, bacterial community composition, and *nosZ* copy numbers negatively correlated with soil N_2_O emission; and nitrate, ammonium, soil total N, and the copy numbers of AOA, AOB, *nirK*, and *nirS* positively correlated with soil N_2_O emission.

**Figure 7:**
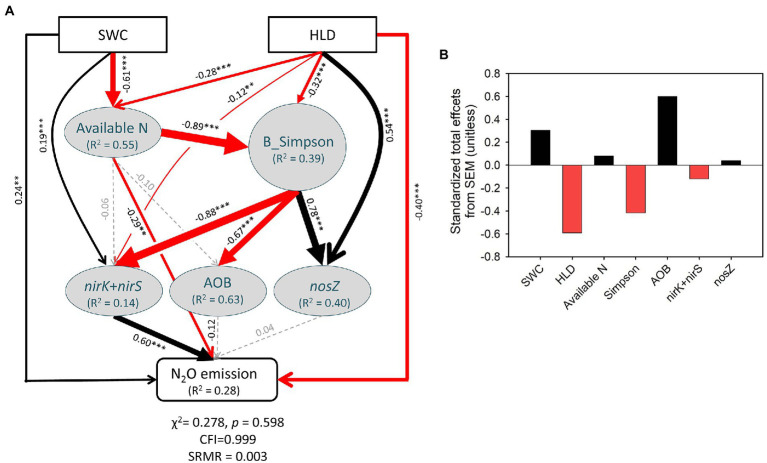
Structural equation modeling (SEM, **A**) depicting the direct and indirect effects of soil water content (SWC) and hyphal length density (HLD) on the N_2_O emission of soil, and standardized total effects (direct plus indirect effects, **B**) of the precipitation, AMF, available N, Bacterial Simpson, and N cycle relative genes on the resistance of N_2_O emission. Significant positive and negative paths (*p* < 0.05) are shown as black and red arrows, respectively, and gray dashed arrows indicate nonsignificant pathways (*p* > 0.05). *p < 0.05, **p < 0.01, and ***p < 0.001.

SEM results provided the direct and indirect effects of precipitation changes and AMF on the emission of soil N_2_O. The SEM results showed that 28% of the variance in soil N_2_O emission could be explained by precipitation and AMF (Figure 7A). Precipitation had a direct positive effect on soil N_2_O emission and indirect positive effects through soil available N, soil bacterial diversity, and functional gene copy numbers. In addition, AMF exerted a directly negative effect on soil N_2_O emission and indirect negative effects through affecting soil bacteria diversity and functional gene copy numbers (Figure 7A). The effects of precipitation changes and AMF on soil N_2_O emission followed opposite trends, as indicated by the standardized total effects from SEM (Figure 7B).

## Discussion

### Precipitation and AMF collectively influence soil N_2_O emissions

Our results showed that precipitation changes and AMF were critical factors to affect soil N_2_O emissions from the semiarid grassland ecosystem. Soil moisture had remarkably positive effects on soil N_2_O emission, which is in agreement with most previous observations in grassland ecosystems (Zhang and Han, 2008; Du et al., 2016; Li et al., 2018). Li et al. (2020) reported that N_2_O emission had been suppressed by 31% by precipitation decrease and increased by 55% in precipitation increase conditions. Because low soil moisture status leads to a decline in the mobility of soil available N elements, on the contrary, high soil moisture status leads to loss of soil available N by leaching, which ultimately leads to changes in soil N_2_O emissions (Li et al., 2020). In addition, O_2_ level variation caused by changes in precipitation was identified as the main driver for activity and alteration in the N_2_O-producing microbial community (Kumar et al., 2020), especially for nitrifiers and denitrifiers. In this study, although reduced precipitation did not alter soil bacterial community composition, the abundance of nitrification and denitrification functional genes was affected by variation in precipitation. This result is consistent with the previous results that NH_3_ oxidation is the principal source of N_2_O at high O_2_ levels by both AOA and AOB communities, while nitrifier denitrification is more dominant under low O_2_ conditions (Sutka et al., 2006).

In addition, our results showed that N_2_O production was reduced in AMF soil, suggesting of AMF mycelium plays a vital role in the mitigation of soil N_2_O emissions under the precipitation changes condition. Several previous studies have demonstrated that AMF might impact N_2_O emission from the soil in the addition of inorganic nitrogen and/or in agro-ecosystems (Bender et al., 2014; Storer et al., 2018; Gui et al., 2021). However, this study highlights the nonlinear response of mycorrhizal fungi in regulating soil N_2_O emission to precipitation gradient reduction in grassland ecosystems. The results supported our second hypothesis that the mycorrhizal response to soil N_2_O emissions has a drought threshold, which was rarely mentioned in previous studies about the effects of AMF on soil N_2_O emissions. This may be attributed to the moderate drought facilitating the function of AMF rather than extreme drought (Li et al., 2019).

### Potential mechanisms of the effects of AMF on N_2_O emission

This study presented for the first time a microbiological regulation mechanism of soil N_2_O emissions by the interaction of AMF and global change factors, i.e., precipitation variability. Firstly, inhibition of soil N_2_O flux by AMF was primarily regulated by the availability of nitrogen concentrations (NH_4_^+^ and NO_3_^−^) in the studied grassland. The concentration of NO_3_^−^ in the AMF-permitted was lower than that in the AMF-excluded treatment, indicating AMF can reduce concentrations of mineral soil N, which is in agreement with the results of an earlier study (Zhang et al., 2015). AMF would be through producing numerous fine hyphae that actively scavenge soil for NH_4_^+^ and NO_3_^−^ and transport N to the plant compartment (Tanaka and Yano, 2005; Whiteside et al., 2009; Veresoglou et al., 2012), suggesting a reduction in the substrate for nitrification thereby inhibiting nitrification rates (Figure 6; Supplementary Figure S5). The presence of AMF significantly increased the content of NH_4_^+^ (Table 1), which is inconsistent with previous findings that AMF either preferentially (Govindarajulu et al., 2005) or exclusively (Tanaka and Yano, 2005) assimilates inorganic N in the form of NH_4_^+^. This could be attributed to (i) the effective acceleration of organic N mineralization by AMF hyphae (Figure 6; Supplementary Figure S5) and (ii) AMF competes with soil microorganisms for NH_4_^+^ leading to a decrease in the utilization of NH_4_^+^ by ammonia-oxidizing bacteria (Storer et al., 2018). Therefore, the effect of AMF on N_2_O emissions depends on the modification of nitrogen substrate concentration by AMF, i.e., the mineralization rate of organic nitrogen and the uptake rate of inorganic nitrogen by the plant-mycorrhizal symbiosis.

Secondly, soil bacteria community composition was determinant for AMF inhibition soil N_2_O emissions in a semiarid grassland. There is some evidence that showed the interaction between AMF and soil microbial community structure in the hyphosphere to explain N_2_O production, but these results are not consistent (Bender et al., 2014; Gui et al., 2021). Gui et al. (2021) found that AMF influenced N_2_O production indirectly by altering the abundance of functional genes, but not by modifying soil chemical properties and soil microbial communities. Our results showed that AMF reduced the abundance of microorganisms associated with N_2_O production (*Nitrospira*, *Anaerolineae*, and *Pyrinomonadaceae*) and increased the abundance of microorganisms associated with N_2_O consumption (Figure 4; Supplementary Figure S3). AMF mycelium has a vital effect on soil microbial communities by regulating the soil microenvironment, i.e., water status (Lazcano et al., 2014), pH, C:N ratio (Govindarajulu et al., 2005; Walder and van der Heijden, 2015), soil structure (Leifheit et al., 2015). Soil microbial community is a key factor in denitrification and nitrification (Veresoglou et al., 2011), which is closely related to both N_2_O production and emission. The changes in microbial community composition may affect the abundance or activity of some microorganisms associated with the N cycle, ultimately leading to a reduction in soil N_2_O production. However, little is known about the mechanisms by which changes in microbial diversity and community composition affect N_2_O production.

Thirdly, AMF-induced microbial functional gene abundance variation plays an important role in AMF-reduced N_2_O emission. We observed a significant negative correlation between hyphal length density (HLD) and the copy numbers of *nirS*, and a positive correlation between HLD and AOB and *nosZ*, which is in agreement with Bender et al. (2014). It has been shown that the genes of *nirS* and *nirK* are used as gene makers for denitrifiers that reduce nitrate to N_2_O (Kandeler et al., 2006) and relative reduction in denitrifying organisms containing the *nosZ* genes can lead to enhanced N_2_O emissions (Philippot et al., 2011). Previous studies revealed that the decreased N_2_O production in the mycorrhizosphere was due to a decrease in nitrification (Veresoglou et al., 2011) and denitrification (Bender et al., 2015). We found direct evidence that AMF suppressed N_2_O production by reducing the net nitrification rate (Supplementary Figure S5). These results suggest that the relationship between AMF and nitrification rates is in agreement with a previous result by Veresoglou et al. (2011). Bender et al. (2014) showed that AMF hypha significantly mitigated N_2_O production by decreasing denitrification in the grassland soil. Our result found that HLD was significantly correlated with gene copy numbers related to denitrification, which might partially explain the indirect reduction in N_2_O emissions by AMF modulation denitrification rates. However, the molecular mechanism of AMF’s effect on N_2_O emissions needs to be further investigated in future work.

## Conclusion

This study highlights that soil moisture status and AMF play key roles in regulating soil N_2_O emission from semiarid grassland. The reduction in precipitation not only directly induced declines in soil water content but also significantly limited soil N_2_O emissions during the growing season. The effects of AMF on soil N_2_O emissions appears to be a consequence of the simultaneous decrease in soil N availability, changes in bacterial community structure, and regulation the abundance of N cycling-related functional genes that we observed. Moreover, our results show that moderate soil moisture decrease or drought would promote the function of AMF in mitigating soil N_2_O emissions by regulating N cycle processes from grassland ecosystems. For the grassland ecosystem, both climate and N cycling are changing in tandem, which has important implications for estimating the regulation of AMF to grassland N cycling process under global climate change in the future. Sustainable management of grassland ecosystems will thereby require a nuanced, mechanistic understanding of soil microorganism interactions between soil moisture status, nutrient status, and greenhouse gas emission.

## Data availability statement

The data presented in the study are deposited in the NCBI repository, accession number PRJNA850526.

## Author contributions

JL contributed to conceptualization, investigation, writing—original draft, and writing—review and editing. BM, XY, NC, and TZo contributed to investigation and writing—reviewing and editing. HC contributed to formal analysis and writing—reviewing and editing. TZn and WS contributed to conceptualization, formal analysis, writing—original draft, and writing—reviewing and editing. All authors contributed to the article and approved the submitted version.

## Funding

This research was funded by the National Natural Science Foundation of China (No. 31870456).

## Conflict of interest

The authors declare that the research was conducted in the absence of any commercial or financial relationships that could be construed as a potential conflict of interest.

## Publisher’s note

All claims expressed in this article are solely those of the authors and do not necessarily represent those of their affiliated organizations, or those of the publisher, the editors and the reviewers. Any product that may be evaluated in this article, or claim that may be made by its manufacturer, is not guaranteed or endorsed by the publisher.
