# Supplementary material for: Suppression of AMF accelerates N2O emission by altering soil bacterial community and genes abundance under varied precipitation conditions in a semiarid grassland
Source: Front Microbiol. 2022 Aug 8;13:961969. doi: 10.3389/fmicb.2022.961969 (PMC9393504; doi:10.3389/fmicb.2022.961969)
Supplement: Supplementary file 1 [file Data_Sheet_1.docx]

**Supplementary material**

**Table S1** Primer pairs and reaction conditions used for 16S rRNA estimation of soil samples.

| **Composition of reaction** | | **volume (µl)** | | **Reaction conditions** |
| --- | --- | --- | --- | --- |
| 10X PCR Buffer | | | 2 | 95 °C for 3 min; 35 cycles of 95 °C for 30 sec, 57 °C for 30 sec, 72 °C for 30 sec; 72 °C for 8 min acquiring after elongation |
| Primer F (10 µM) | | | 0.5 |  |
| Primer R (10 µM) | | | 0.5 |  |
| dNTP（each 10 mM） | | | 0.5 |  |
| MgCl2 (25mM) | | | 2 |  |
| Taq Plus DNA Polymerase（5 U/μl） | | | 0.5 |  |
| Template (DNA) | | | 2 |  |
| ddH_2_0 | | | 17 |  |
| Total | | | 25 |  |
| **Target** | **Primer** | | **Sequence** | |
| Bacterial 16S rRNA | 338F | | 5'- ACTCCTACGGGAGGCAGCA-3' | |
|  | 806R | | 5'- GGACTACHVGGGTWTCTAAT-3' | |

**Table S2** Primer pairs and reaction conditions used for gene copy number estimation of soil samples.

| **Composition of reaction** | | | **volume (µl)** | | | | | | |
| --- | --- | --- | --- | --- | --- | --- | --- | --- | --- |
| SybrGreen qPCR Master Mix | | | 5 | | | | | | |
| Primer F (10 µM) | | | 0.2 | | | | | | |
| Primer R (10 µM) | | | 0.2 | | | | | | |
| ddH_2_0 | | | 3.6 | | | | | | |
| Template (DNA) | | | 1.0 | | | | | | |
| Total | | | 10 | | | | | | |
| **Reaction conditions** | | | | | | | | | |
| Thermal Cycler | | Times and Temperatures | | | | | | Dissociation | |
|  |  | Initial Steps | | Each of 45 cycles | | | | Accoding to instrument guidelines | |
|  |  |  |  | Melt | | Anneal/Extend | |  |  |
| LightCycler480 II | | HOLD | | CYCLE | | | |  |  |
|  |  | 3 min 95°C | | 5 s 95°C | | 30 s 60°C | |  |  |
| **Target** | **Primer** | | | | **Sequence** | |  |  |  |
| *amo*A | *amoA*F | | | | 5′-STAATGGTCTGGCTTAGACG-3′ | | | | |
|  | *amoA*R | | | | 5′-GCGGCCATCCATCTGTATGT-3′ | | | | |
| *amoB* | AOB-F | | | | 5’-GGGGTTTCTACTGGTGGT-3’ | | | | |
|  | AOB-R | | | | 5’-CCCCTCKGSAAAGCCTTCTTC-3’ | | | | |
| *nirK* | *nirK*F | | | | 5’-ATCATGGTSCTGCCGCG-3’ | | | | |
|  | *nirK*R | | | | 5’-GCCTCGATCAGRTTGTGGTT-3’ | | | | |
| *nirS* | 4QF | | | | 5’-GTSAACGYSAAGGARACSGG-3’ | | | | |
|  | 6QR | | | | 5’-GASTTCGGRTGSGTCTTGA-3’ | | | | |
| *nosZ* | *nosZ*-F | | | | 5′-CGYTGTTCMTCGACAGCCAG-3′ | | | | |
|  | *nosZ*-R | | | | 5’-CGSACCTTSTTGCCSTYGCG-3’ | | | | |

**
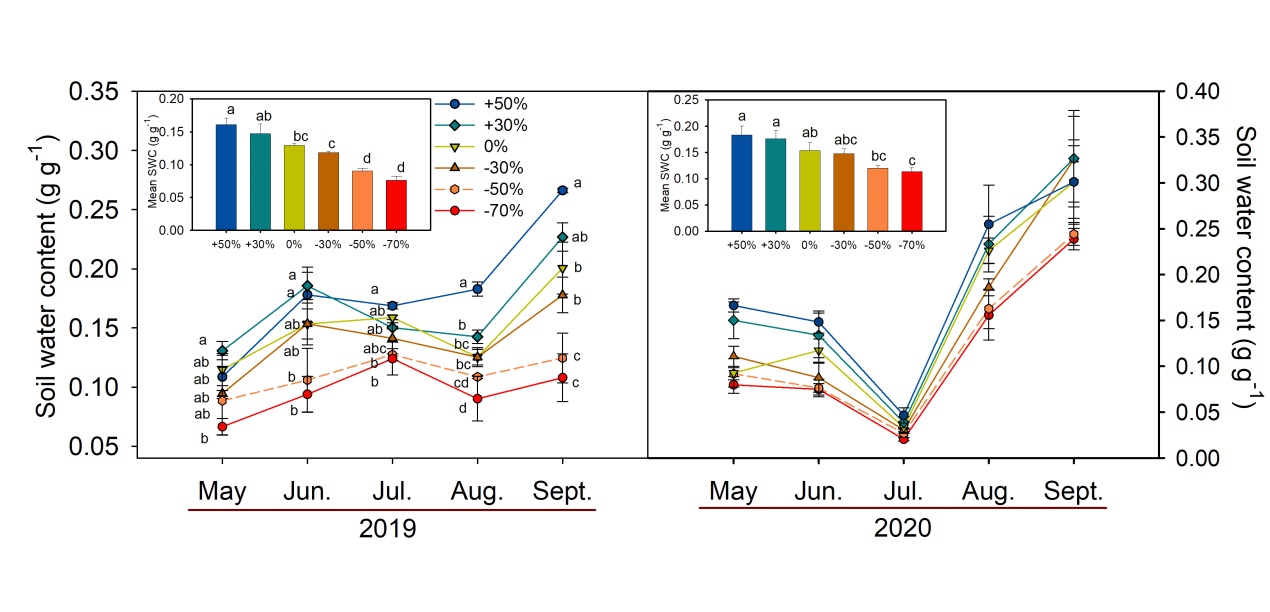
**

**Figure S1** Seasonal dynamics of soil water content (SWC, g/g) and seasonal mean soil water content at 0-10 cm depth of each precipitation treatment in 2019 (A) and 2020 (B). Different lowercase letters indicate significant differences (*P* < 0.05) among the precipitation treatments. Data are reported as mean ± 1 SE (n = 4).

**
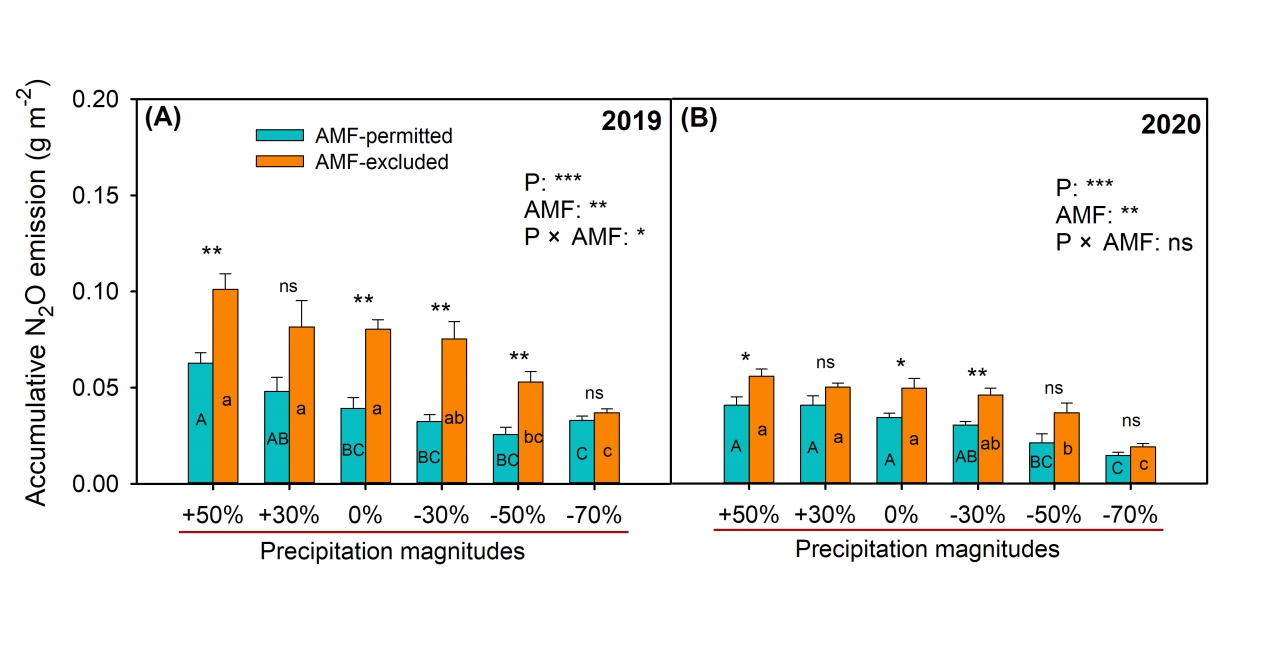
**

**Figure S2** Accumulative N_2_O emission of AMF-permitted and AMF-excluded under the six precipitation conditions in 2019 (A) and 2020 (B). ****P* < 0.001, ***P* < 0.01, and **P* < 0.05 indicates differences between AMF-permitted and AMF-excluded. Data are reported as mean ± 1 SE (n = 4).

**
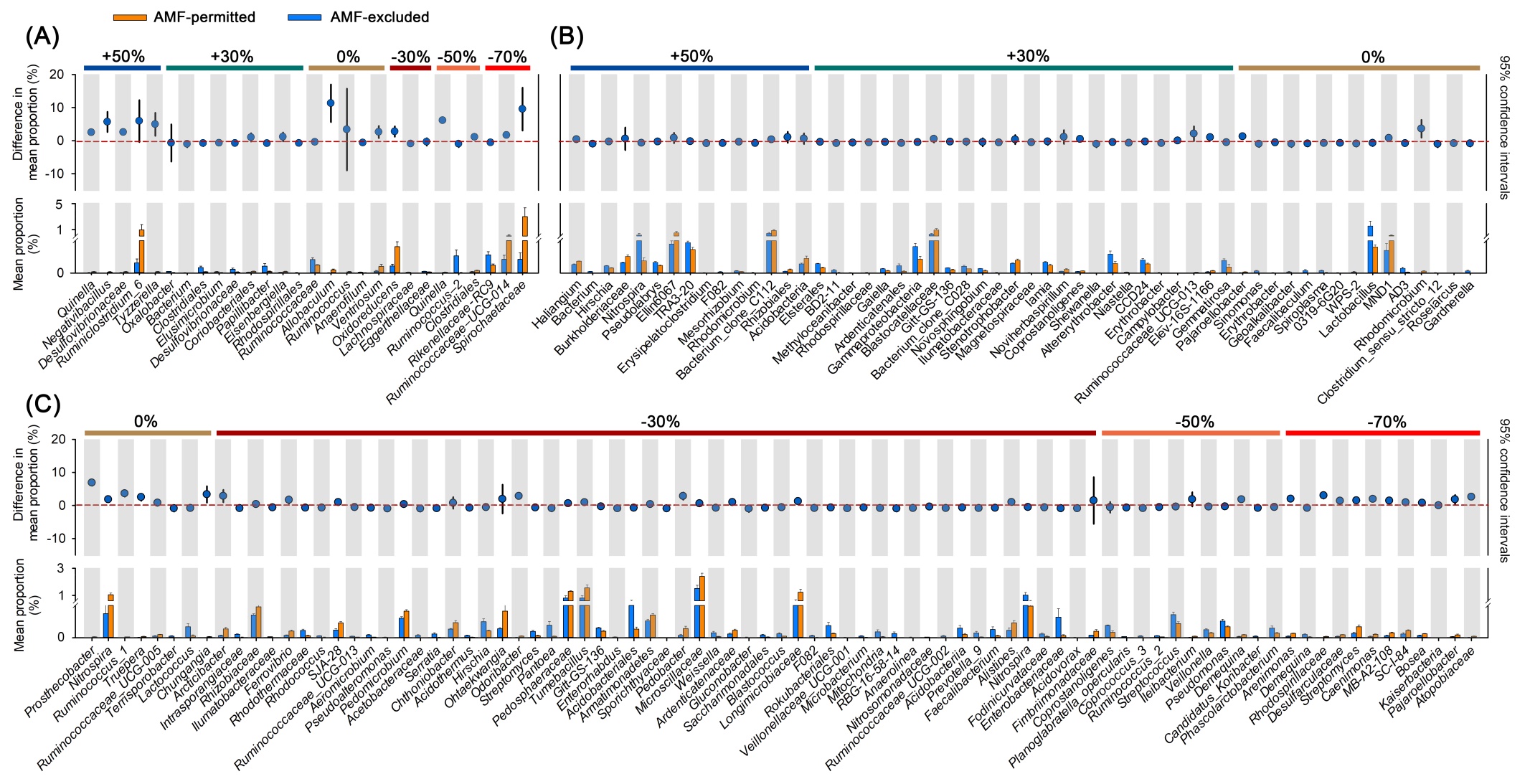
**

**Figure S3** Variation analysis of bacteria community at the genus level in AMF-permitted as comparison of that in AMF-excluded under the different precipitation conditions in 2019 (A) and 2020 (B &C).

**
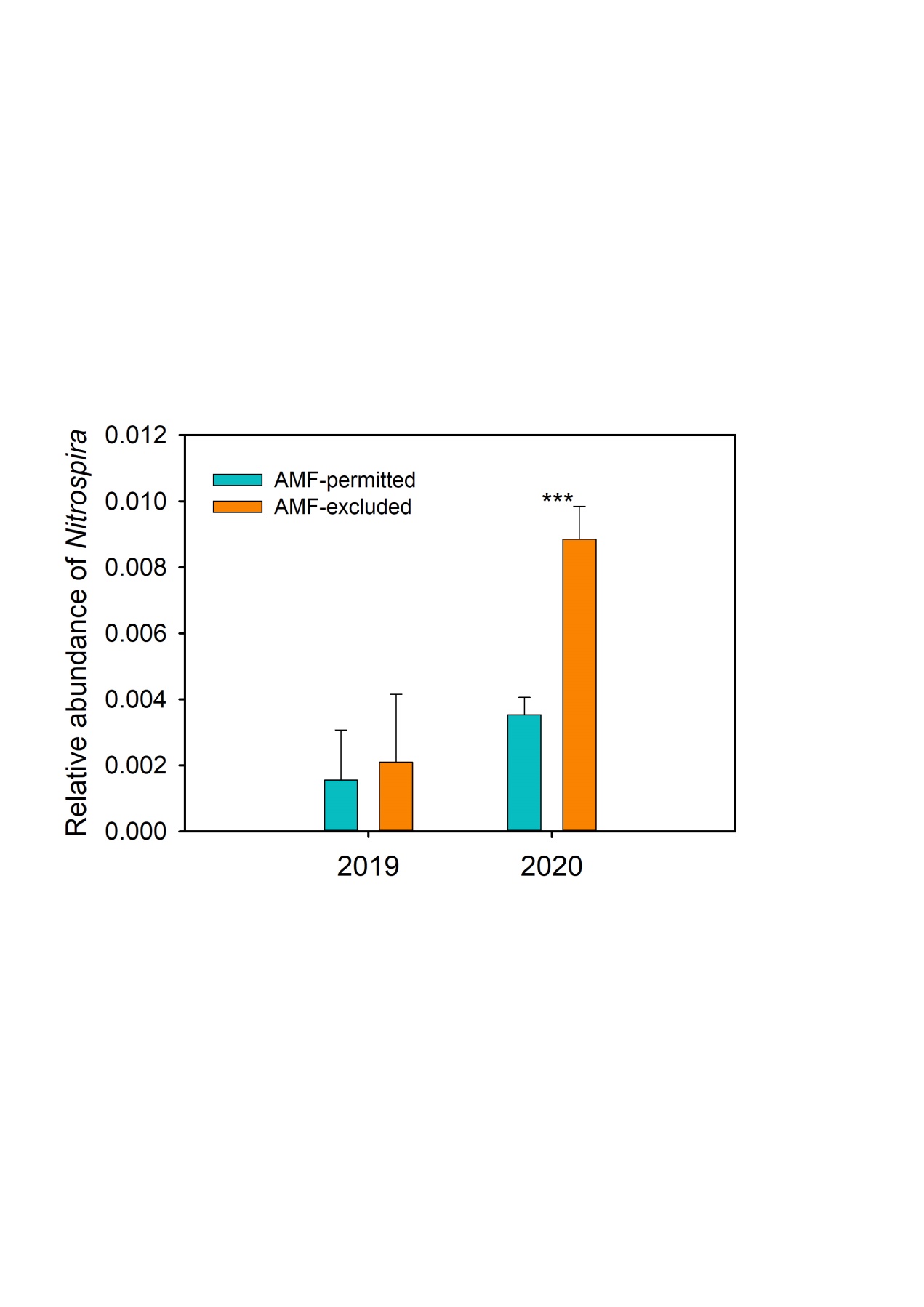
**

**Figure S4** Relative abundance of *Nitrospira* of AMF-permitted and AMF-excluded under the six precipitation conditions in 2019 and 2020. ****P* < 0.001 indicates differences between AMF-permitted and AMF-excluded. Data are reported as mean ± 1 SE (n = 4).


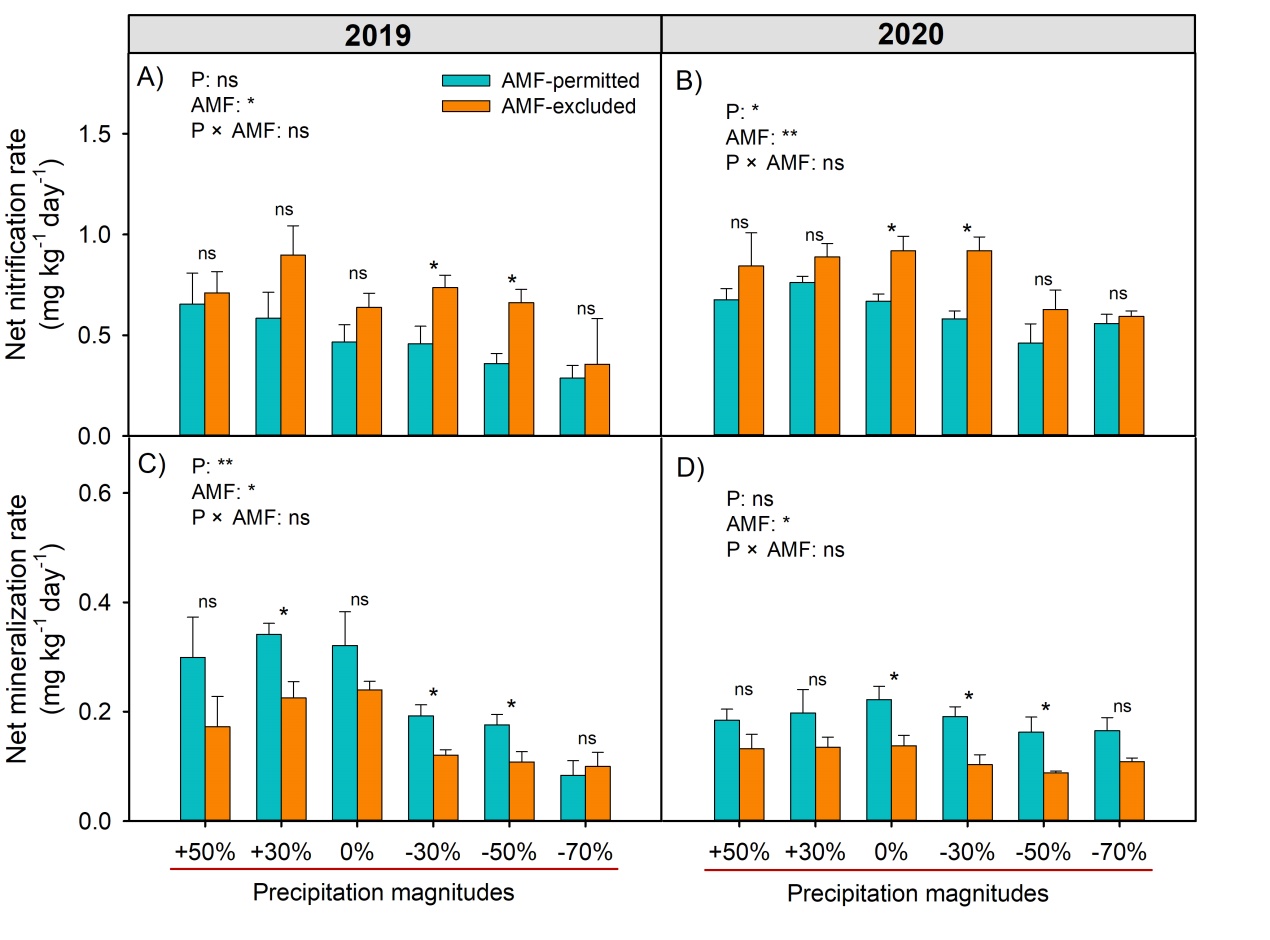


**Figure S5** Effects of AMF on soil net nitrification rate (A, B) and net mineralization rate (C, D) under different precipitation conditions in 2019 and 2020. Asterisks indicate significant differences between AMF-permitted and AMF-excluded under the same precipitation conditions (**P* < 0.05, **0.001 < *P* < 0.01 ****P* < 0.001 and ns *P* > 0.05). Data are reported as mean ± 1 SE (n = 4).
